# Stand-Alone or Combinatorial Effects of Grafting and Microbial and Non-Microbial Derived Compounds on Vigour, Yield and Nutritive and Functional Quality of Greenhouse Eggplant

**DOI:** 10.3390/plants11091175

**Published:** 2022-04-27

**Authors:** Leo Sabatino, Beppe Benedetto Consentino, Georgia Ntatsi, Salvatore La Bella, Sara Baldassano, Youssef Rouphael

**Affiliations:** 1 Department of Agricultural, Food and Forest Sciences (SAAF), University of Palermo, Viale delle Scienze, Ed. 5, 90128 Palermo, Italy; beppebenedetto.consentino@unipa.it (B.B.C.); salvatore.labella@unipa.it (S.L.B.); 2 Laboratory of Vegetable Production, Department of Crop Science, Agricultural University of Athens, 11855 Athens, Greece; 3 Department of Biological, Chemical and Pharmaceutical Sciences and Technologies, University of Palermo, 90128 Palermo, Italy; sara.baldassano@unipa.it; 4 Department of Agricultural Sciences, University of Naples Federico II, 80055 Portici, Italy; youssef.rouphael@unina.it

**Keywords:** seaweed extracts, functional quality, *Trichoderma atroviride*, *Solanum melongena*, *Solanum torvum*, sustainable horticulture

## Abstract

The current research investigated the effects of endophytic fungi such as *Trichoderma atroviride* (Ta) or *Ascophyllum nodosum* seaweed extract (An) and their combination on growth, yield, nutritive and functional features, and mineral profile of ‘Birgah’ F_1_ eggplant either ungrafted, self-grafted or grafted onto the *Solanum torvum* rootstock. Eggplant exposed to An or An+Ta had a significant increase in root collar diameter 50 days after transplanting (RCD_50_), total yield (TY), marketable yield (MY), ascorbic acid (AA) content, Mg, Cu, and Zn concentration, and a reduction in glycoalkaloids (GLY) compared with the control. Furthermore, grafted plants had a higher TY, MY, number of marketable fruits (NMF), RCD_50_, AA, Cu, and Zn and a lower SSC, GLY, and Mg than the ungrafted plants. The combination of grafting and An+Ta significantly improved mean weight of marketable fruits (MF), plant height 50 days after transplanting (PH_50_), number of leaves 50 days after transplanting (NL_50_), fruit dry matter (FDM), chlorogenic acid (ClA), proteins, and K and Fe concentration. This combination also produced fruits of high premium quality as evidenced by the higher AA and ClA concentration, the lower GLY concentration, and an overall improved mineral profile.

## 1. Introduction

The fast growth of the world population will cause a boost in the world-wide food requirement, estimated to double in 2050 [1]. Enhancing the agricultural production seems to be the only approach to face the rapid increase in food need [1]. This strategy demands the use of high-input resources such as protected cultivation systems, which, however, can negatively affect the agroecosystem [2]. Indeed, greenhouse techniques use a high quantity of synthetic fertilizers, which can cause damage to both the environment and human health [3,4]. The leafy and fruiting vegetable production sector can adopt specific agronomic practices to minimize its impact on the ecosystem [5,6,7,8]. In this scenario, a promising strategy, which could encourage a change from a fertilizers-intensive agriculture to an eco-friendlier system, is the integrated use of biostimulants [9,10,11,12,13,14,15,16]. Biostimulants are products that can stimulate plant growth and development due to several bioactive properties of their compounds [9]. Biostimulants can be grouped as either microbial (e.g., *Trichoderma* spp., arbuscular mycorrhiza fungi, and growth-promoting bacteria) or non-microbial (e.g., seaweed extract, vegetal and animal-based protein hydrolysates, humic and fulvic acids, and silicon) [10,17,18,19]. Among the former, fungi, such as *Trichoderma*, are often employed in agriculture to enhance yield, quality, nutrient uptake, and plant growth [20,21]. Moreover, *Trichoderma* are associated with the biocontrol of diseases/pests and the stimulation of plant photosynthesis and metabolism [22,23]. Within the non-microbial biostimulant group, brown algae extracts are the most adopted in horticulture, as they contain a high quantity of signalling molecules [24,25]. The increase in production and quality driven by seaweed extract application has been related to the elicitation of carbon and nitrogen metabolic ways, the Krebs cycle and glycolysis and to the change of root architecture, which can promote mineral assimilation and accumulation [26].

Herbaceous grafting is a suitable propagation practice to address the negative impact of several biotic and/or abiotic cultivation factors that can totally or partially compromise yield and fruit quality of fruiting vegetables [27,28]. Moreover, there are reports of fruit yield and quality improvement of various grafted vegetables grown under optimal or sub-optimal conditions [29,30]. In this respect, the employment of grafted vegetables has become a common, eco-friendly, and profitable horticultural practice [31].

Eggplant (*Solanum melongena* L.) belongs to the solanaceous, a huge plant family embracing approximately 3000 species. As reported by Daunay [32], *S. melongena* was introduced to the Mediterranean Basin in the 7th to 8th centuries CE. Currently, eggplant is one of the most eaten vegetables worldwide. The eggplant global production is approximately 50 Mt, and it is mostly concentrated in Asia. Egypt, Turkey, and Italy are the leading producers in the Mediterranean Basin [33]. Sicily, where eggplant is conventionally cultivated in both a protected environment (fall-winter period) and open fields (spring-summer period) [34], with an almost continuous production throughout the year, is considered a secondary centre of differentiation [35] of this crop.

The current research detects some standpoints to design and develop new application protocols to valorize the synergistic effects of different biostimulant categories to render agriculture more sustainable and resilient.

Grafting and biostimulants are easy, effective, and tangible practices applied to produce fruiting vegetables. Nevertheless, as plant reaction to grafting and biostimulants is modulated by genotype and cultivation conditions, an exhaustive study to evaluate techniques and dosages is crucial. To the best of our knowledge, there is no literature on the impact of the combined use of grafting and biostimulants on fruit production and quality features of eggplant. Thus, the scope of the study was to assess the interactive effect of grafting and of microbial and non-microbial biostimulants on plant growth, production, and qualitative parameters of eggplant grown under protected cultivation.

## 2. Results

### 2.1. Plant Performance and Production

**Figure 1:**
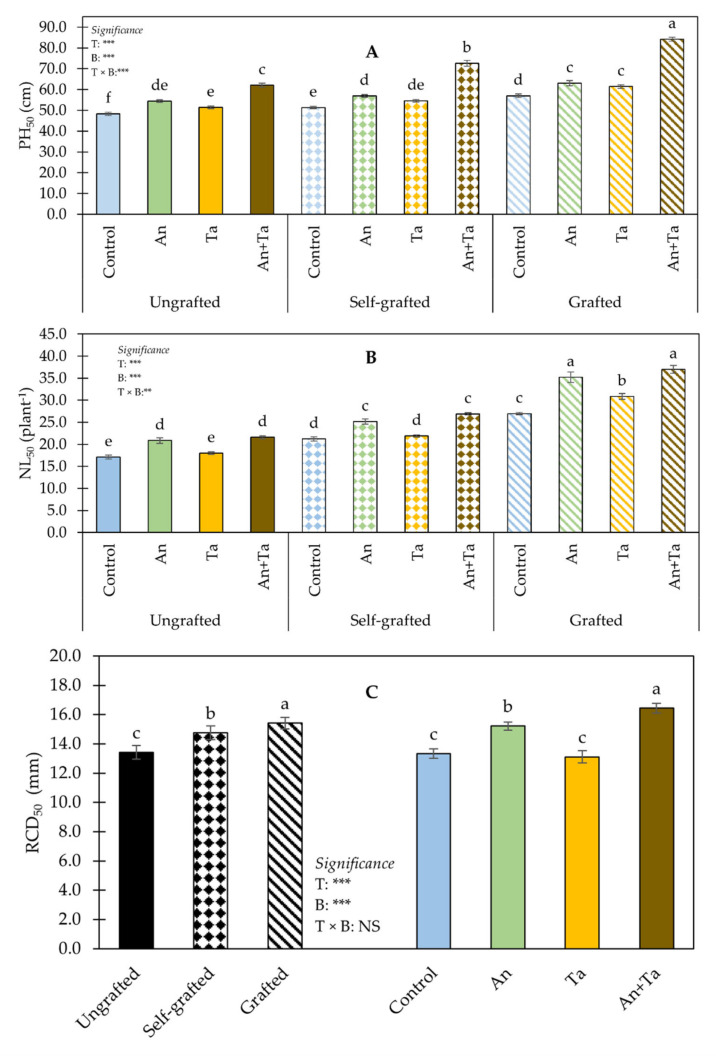
Effect of plant type (T) and biostimulant (**B**) on plant height 50 day after transplant (PH_50_) (**A**), number of leaves 50 days after transplant (NL_50_) (**B**) and root collar diameter 50 days after transplant (RCD_50_) (**C**) of ‘Birgah’ F_1_ eggplant. Bars show means ± SE. Bars with same letter are not significantly different accordingly to Tukey’s test at *p* ≤ 0.05. Significance: NS non-significant; ** significant at *p* ≤ 0.01; *** significant at *p* ≤ 0.001. An: *Ascophyllum nodosum*; Ta: *Trichoderma atroviride*.

Data on plant growth parameters are presented in Figure 1.

ANOVA for PH_50_ revealed a significant influence of the interaction plant type (T) × biostimulant (B) (Figure 1A). Plants grafted onto *S. torvum* rootstock and supplied with both biostimulants (An+Ta) had the highest height, followed by those self-grafted and treated with both An and Ta. The lowest PH_50_ values were recorded in ungrafted non-biostimulated plants.

ANOVA for NL_50_ exhibited a significant effect of the T × B interaction (Figure 1B). ‘Birgah’ F_1_ grafted eggplants treated with An or An+Ta had the highest NL_50_, followed by those grafted and supplied with Ta. The lowest NL_50_ was found in untreated plants (control) and in ungrafted plants treated with Ta.

ANOVA for RCD_50_ did not display a significant effect of the T × B interaction (Figure 1C). Regardless of the biostimulant application, grafted plants had the highest RCD_50_ value, whereas ungrafted plants had the lowest. Irrespective of T, plants treated with An+Ta showed the highest RCD_50_ values, followed by those exposed to An. The lowest values were detected in control plants and in those exposed to Ta.

**Figure 2:**
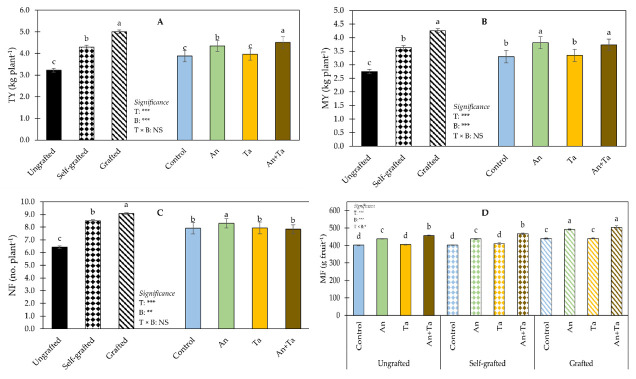
Effect of plant type (T) and biostimulant (**B**) on total yield (TY) (**A**), marketable yield (MY) (**B**), number of marketable fruits (NF) (**C**) and mean weight of marketable fruits (MF) (**D**) of ‘Birgah’ F_1_ eggplant. Bars show means ± SE. Bars with same letter are not significantly different accordingly to Tukey’s test at *p* ≤ 0.05. Significance: NS non-significant; * significant at *p* ≤ 0.05; ** significant at *p* ≤ 0.01; *** significant at *p* ≤ 0.001. An: *Ascophyllum nodosum*; Ta: *Trichoderma atroviride*.

Yield features are shown in Figure 2.

No significant T × B interaction was found for TY (Figure 2A). Grafted plants revealed the highest TY, followed by self-grafted plants, whereas ungrafted plants had the lowest TY. Irrespective of the type of plant, plants treated with An+Ta revealed the highest TY, followed by those supplied with An. Untreated plants and those treated with Ta had the lowest TY.

ANOVA for MY did not show a significant effect of the T × B interaction (Figure 2B). Regardless of the biostimulant supply, data on MY followed the trend established for TY. Disregarding the type of plant, those treated with An or An+Ta showed the highest MY, followed by the non-treated (control) or supplied with Ta.

For NF, ANOVA did not reveal a significant effect of the T × B interaction (Figure 2C). Notwithstanding the biostimulant application, data on NF followed the same tendency recorded for TY and MY. Plants subjected to An application had the highest NF, whereas those exposed to Ta revealed the lowest values.

ANOVA for MF revealed a significant effect of the interaction between T and B (Figure 2D). Plants grafted onto *S. torvum* and treated with An or An+Ta showed the highest MF, followed by those ungrafted or self-grafted and supplied with An+Ta. The lowest MF was recorded in the combinations ungrafted × control, ungrafted × Ta, self-grafted × control, and self-grafted × Ta eggplant.

### 2.2. Nutritional and Functional Fruit Traits

Data on fruit quality and mineral profile are presented in Table 1 and Table 2. For FDM, firmness, ClA, TA, proteins, K, and Fe, ANOVA displayed a significant effect of the T × B interaction (Table 1). The combinations grafted plants × Ta and grafted plants × An+Ta had the highest FDM. The lowest values were recorded in the combinations of ungrafted plants × control, ungrafted plants × Ta, and self-grafted plants × control eggplants.

All plant types (ungrafted, self-grafted, or grafted), non-biostimulated or inoculated with Ta, showed the highest firmness. Supplying grafted eggplants with An+Ta resulted in reduced fruit firmness compared to the other treatments.

Grafted plants supplied with An or An+Ta had the highest ClA, protein, and Fe concentrations, followed by the combinations of grafted × control and grafted × Ta. The lowest ClA, protein, and Fe concentrations were detected in fruits from ungrafted × control, ungrafted × Ta, self-grafted × control, and self-grafted × Ta eggplant combinations.

Self-grafted plants treated with An or An+Ta showed the highest TA concentration, followed by the combinations of ungrafted × An, ungrafted × An+Ta, self-grafted × control, and self-grafted × Ta eggplants. The lowest TA concentration was detected in fruits from untreated plants or Ta inoculated grafted plants.

Grafted plants treated with both biostimulants showed the highest fruit K concentration, followed by the grafted and inoculated with Ta eggplants. The lowest fruit K concentration was recorded in fruits from the combination of ungrafted × control eggplants.

ANOVA for SSC, AA, GLY, P, Ca, Mg, Cu, and Zn did not display a significant effect of the T× B interaction (Table 2).

Regardless of the biostimulant supply, ungrafted plants showed the highest fruit SSC, whereas grafted plants exhibited the lowest values. Conversely, independent of the type of plant, biostimulants did not affect fruit SSC.

Grafted eggplants showed the highest fruit AA, Cu, and Zn concentrations, whereas fruits from self-grafted and ungrafted plants had the lowest ones. Irrespective of the type of plant, fruits from plots treated with An and An+Ta had the highest AA, Cu, and Zn concentrations. The lowest fruit AA, Cu, and Zn concentrations were recorded in the control plants and those supplied with Ta.

Ungrafted and self-grafted plants showed the highest fruit GLY concentration, whereas grafted plants had the lowest one. Without regard to the type of plant, both control and Ta inoculated plants displayed the highest fruit GLY concentration. The lowest GLY values were recorded in fruits from plots treated with An or An+Ta.

ANOVA showed that both main factors did not significantly affect fruit P and Ca content.

Regardless of the biostimulant, the fruit from ungrafted and self-grafted plants had the highest Mg concentration. Irrespective of the type of plant, fruits from plots treated with An+Ta exhibited the highest Mg content, followed by those from plots sprayed with An, which in turn had a higher Mg concentration than those from plots inoculated with Ta. The lowest fruit Mg concentration was detected in the control eggplants.

### 2.3. Heat Map Analysis of All Eggplant Traits

**Figure 3:**
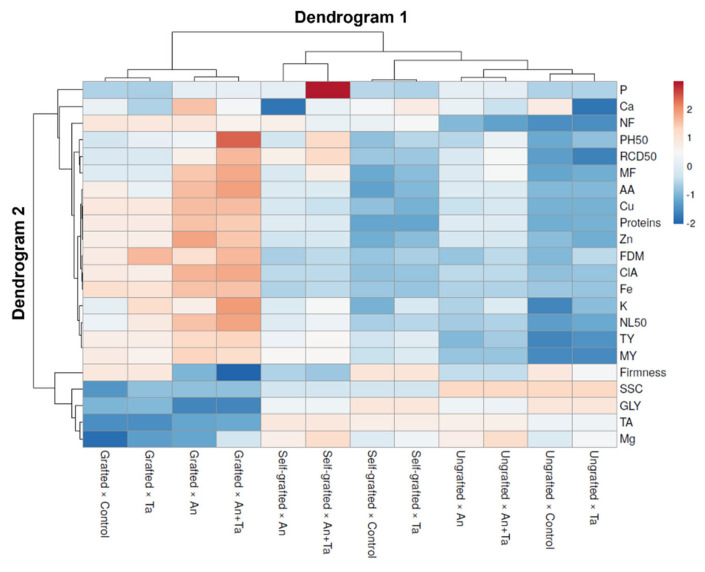
Heat map analysis including all eggplant plant features in response to grafting and biostimulant application. The heat map was realized via the https://biit.cs.ut.ee/clustvis/ (accessed on 1 March 2022) online program package. An: *Ascophyllum nodosum*; Ta: *Trichoderma atroviride*.

A heat-map analysis of all tested parameters, comprising growth, yield, and fruit quality traits, was performed to produce a graphical evaluation of the respective experimental factors effect on ‘Birgah’ F_1_ eggplant (Figure 3).

Heat-map analysis revealed two dendrograms. Dendrogram 1 was placed on the top, encompassing the groupings of type of plant and biostimulant, and Dendrogram 2, sited on the left, involving the studied parameters that affected this distribution. Dendrogram 1 showed two main groups; the group on the left side involves all combinations with grafted plants (grafted × control, grafted ×Ta, grafted × An, and grafted × An+Ta). The other, located on the right, includes all combinations with ungrafted and self-grafted plants. Explicitly, in the left cluster of Dendrogram 1, the grafted × control and grafted × Ta combinations were separated from the combinations grafted × An and grafted × An+Ta due to the lower P, Ca, PH_50_, RCD_50_, MF, AA, Cu, proteins, Zn, ClA, Fe, K, NL_50_, TA, and Mg. The cluster on the left comprised the grafted × control and grafted × Ta combinations. Inside this cluster, the grafted × control combination was evidently parted by lower PH_50_, FDM, K, NL_50_, SSC, and Mg. The cluster on the right enclosed the grafted × An and grafted × An+Ta combinations. Within this group, the grafted × An+Ta combination was separated by a higher PH_50_, RCD_50_, MF, AA, FDM, K, NL_50_, and Mg.

On the right side of Dendrogram 1, two chief clusters were documented. The cluster on the left incorporated the combinations self-grafted × An and self-grafted × An+Ta, divided from the self-grafted control plants or self-grafted plants treated with Ta and all combinations involving the ungrafted plants, that had, particularly, a higher P, NF, PH_50_, RCD_50_, MF, K, NL_50_, TY, and MY, but a lower firmness, SSC, GLY, TA, and Mg. The cluster on the left included the self-grafted × An and self-grafted × An+Ta combinations; the self-grafted × An combination was separated from the self-grafted × An+Ta combination by lower P, Ca, PH_50_, RCD_50_, MF, TA, and Mg. The cluster on the right comprised self-grafted untreated and Ta inoculated plants and all the combinations including ungrafted plants. In this group, the self-grafted × control and self-grafted × Ta combinations were clearly separated from all the ungrafted combinations by the lower P, PH_50_, SSC, and Mg. The left side of this group included the self-grafted × control and self-grafted × Ta combinations; the self-grafted × control combination was separated due to the lower Ca, PH_50_, MF, AA, Zn, and K. The right side of the cluster included all the ungrafted eggplant combinations. The ungrafted × An and ungrafted × An+Ta combinations were divided by higher P, PH_50_, RCD_50_, MF, AA, Cu, proteins, Zn, ClA, K, NL_50_, TY, MY, and Mg. Inside this cluster, ungrafted An treated plants were parted by lower PH_50_, RCD_50_, MF, FDM, K, TY, and Mg. The right side of this group enclosed the ungrafted × control and ungrafted ×Ta combinations; particularly, the ungrafted × control combination is divided by lower PH_50_, MF, K, and Mg.

## 3. Discussion

Currently, *Trichoderma* is recognized as a valuable plant biostimulant due to its direct and/or secondary benefits related to the *Trichoderma*-plant molecular crosstalk and interchange of various elements and small peptides. These mechanisms elicit numerous plant responses, mineral absorption improvement, and tolerance to mineral distress in vegetables [36,37]. At the same time, seaweed extract is an additional conspicuous category of biostimulants that has displayed positive effects on overall plant performance, mineral uptake, and tolerance to different abiotic stresses affecting vegetable crops [9,10,26]. In addition to biostimulants, herbaceous grafting is a profitable tool for safeguarding and augmenting production, nutritional, and functional features of fruiting vegetables under optimal, sub-optimal, or unfavourable cultivation conditions [7,28,38,39,40,41]. Thus, in the current research, grafting and biostimulant effects—alone or combined—were appraised on plant growth and productivity, as well as fruit nutritional and nutraceutical components of ‘Birgah’ F_1_ eggplant. Results showed that grafting combined with An or An+Ta treatments significantly increased plant growth and yield traits. Similarly, *Ascophyllum nodosum* extract application increased spinach productivity [42] and sweet pepper growth [43]. Moreover, a significant increase of growth parameters of eggplant supplied with boric acid and seaweed extract was recorded by El-Gawad and Osman [44]. The increase of growth and yield of vegetable crops seems to be related to the cytokinins and auxin precursors included in *A. nodosum* extract, which stimulate cell division of plant tissues [42,44]. Moreover, there are reports that the higher plant yield of An-treated plants could be associated with the polysaccharides (alginates, fucoidan, and laminarins) in the *A. nodosum* seaweed extracts, which in turn trigger endogenous hormones homeostasis [45,46]. Blunden and Gordon [47] found that *A. nodosum* extracts contain betaines and betaine-like compounds. These components could lead to improved leaf chlorophyll and photosynthetic rates, resulting in plant productivity increase.

*Thricoderma* inoculation was previously demonstrated to have positive influences on tomato yield and growth [48]. Our findings are supported by Rouphael et al. [49], who found a yield increase of inoculated lettuce plants treated with *Thricoderma virens* and a biopolymer-based biostimulant. These results could be linked to the release of molecules with hormone-like activity, which upsurge plant nutrient bioavailability, uptake, translocation, and accumulation [22,50]. Moreover, it has been confirmed that *Trichoderma* inoculation stimulates root growth and modifies its structure, which in turn enhances nutrient uptake [23,51]. In this study, grafting onto *S. torvum* significantly increased eggplant productivity, thereby confirming the results of Consentino et al. [41]. The positive influence of grafting on plant growth and production was also described in tomato [40]. These results were associated to a boosted mineral and water uptake prompted by grafting [52,53]. In our study, we may assume that the growth and yield increase was related to the synergetic effect of grafting and biostimulants.

Findings revealed that FDM was highest in grafted plants treated with Ta or An+Ta, whereas, in ungrafted plants An did not significantly improve FDM. Thus, our data partly agree with those appraised by Rouphael at al. [42], who observed that *A. nodosum*-based extract did not significantly affect dry matter in spinach plants, although an increasing trend was recorded. Conversely, our findings highlighted a positive impact of *Trichoderma* inoculation, alone or combined with An. These results are corroborated by those of Rouphael et al. [49], who showed an upsurge of plant dry biomass in *Trichoderma*-treated plants. Thus, we may hypothesize that the FDM increase of grafted Ta and An+Ta treated plants was due to their enhanced plant nutritional status.

Seaweed extract alone or combined with *Trichoderma* reduced fruit firmness. These results overlap with those of Ali et al. [54], who found that *A. nodosum* extract decreases skin and pulp firmness in tomato. Concomitantly, in our study grafting improved fruit firmness. This outcome agrees with that of Sabatino et al. [30] who, by studying the combined effect of grafting and arbuscular mycorrhiza on eggplant performance and fruit quality, found that fruits from untreated controls have a lower firmness than those from *S. torvum* grafted plants. Therefore, combining grafting with the application of both biostimulants resulted in a relevant synergistic effect.

Grafted eggplants treated with An or An+Ta had the highest fruit ClA and AA concentrations. A considerable increase in total phenols and ascorbic acid in lettuce plants treated with *A. nodosum* extract, as observed in this study in eggplant, was reported by Rouphael et al. [42]. Ertani et al. [55] and Rouphael et al. [42] report that the enhanced biosynthesis of these metabolites could be connected both to the direct and/or indirect influences of the key enzyme activity included in phytochemical homeostasis and to the modification of plant nutritional status. Comparable results were accomplished by Fan et al. [56] who, by assessing the influence of *A. nodosum* extract application on spinach phenolic antioxidant content, detected an increase in flavonoids compared to the non-biostimulated plants. Furthermore, as reported by Fan et al. [57], the enhancement in biosynthesis of bioactive molecules could be intermediated via the stimulation of chalcone isomerase, which is a key enzyme in the flavanone precursor biosynthesis. Our data highlighted that combining Ta and An trigger a high production of AA and ClA and—as speculated by Rouphael et al. [42]—glutathione, which is a metabolite that acts synergistically with AA to make an antioxidant plant response, protecting the plants from the reactive oxygen species (ROS) [58,59].

Results indicated that grafting significantly increased fruit ClA concentration. This is in contrast with the finding of Darré et al. [60] who, by studying the effects of a cold-tolerant rootstock on eggplant fruit susceptibility, found a reduction of ClA in grafted plants. However, our data concur with those reported by Sabatino et al. [61], who noticed that grafting can increase or reduce total phenolics in relation to the scion-rootstock combination. This is also confirmed by other reports [62,63]. Data showed that fruits from grafted plants had a higher AA content than those from ungrafted or self-grafted plants. These findings are in line with those of Sabatino et al. [30], who observed an increase of AA in fruit from plants grafted onto *S. torvum* compared with those from ungrafted or self-grafted plants. In this respect, Oztekin et al. [64] pointed out that grafting does not significantly influence AA concentration in tomato fruits. These divergent outcomes could be ascribed to the diverse rootstock adopted [65].

The outcomes of this research revealed that self-grafted plants treated with An or An+Ta had the highest fruit TA concentration. These results are coherent with those of Roussos et al. [66] who, by investigating the influence of *A. nodosum* application on strawberry fruit quality, observed that plants treated with the biostimulants had a higher TA content than the untreated ones. Similarly, by studying the effects of genetic factors and *A. nodosum* application on eggplant production and quality, Pohl et al. [67] found that biostimulant supply significantly enhances fruit total anthocyanin concentration.

The use of *S. torvum* as rootstock resulted in reduced TA concentration, similar to the results of Consentino et al. [41] and Sabatino et al. [30]. As it is well known that anthocyanins biosynthesis and accumulation is strongly related to light exposure [68,69] and appraising that grafting technique boosted plant vigour parameters, we can assume that the TA reduction in grafted plants was the result of more shaded fruits.

Overall, our results showed that grafting combined with An or An+Ta significantly increased proteins and mineral concentration in eggplant fruits with the exception of P and Ca. According to Ali et al. [54], Rouphael et al. [42] and Ertani et al. [46], *A. nodosum* extract enhanced mineral concentration in tomato fruits, spinach, and maize, respectively. The recorded upsurge of mineral concentration in fruits from plants treated with seaweed extract was also reported by several other authors [70,71,72]. This effect seems to be due to an up-regulation of genes involved in mineral transporters and/or in the stimulation of root development. Furthermore, grafting significantly augmented fruit mineral concentration, except for Mg. These findings are corroborated by those of Sabatino et al. [30], who reported a higher mineral concentration in fruits from grafted plants compared to those from self-grafted or ungrafted ones. According to Oztekin et al. [64], the higher mineral concentration observed in fruits from grafted plants could be ascribed to an improved mineral and water absorption of grafted plants. Interestingly, our data also pointed out that the combined application of grafting and An+Ta positively influenced mineral profile and protein content in eggplant fruits, suggesting that both experimental factors had a synergetic action.

In our study, SSC was not affected by biostimulant treatments, whereas it was reduced by grafting. Consentino et al. [41] and Sabatino et al. [30] also found an SSC reduction in fruits from plants grafted onto *S. torvum* compared to those from ungrafted or self-grafted plants. A similar pattern of results was obtained by Riga [73] on tomato. Results showed that both An and An+Ta significantly reduced GLY fruit concentration. Similarly, Mystkowska [74] found a lower glycoalkaloids concentration in biostimulated potato plants when compared to the untreated control. The use of *S. torvum* as rootstock, as well, resulted in reduced glycoalkaloids concentration. As Papathanasiou et al. [75] reported that unfavourable growing conditions and cultivation techniques enhance glycoalkaloids concentration in potato, we might assume that grafted plants supplied with An or An+Ta were less stressed than others due to a lower content of GLY. These considerations are entirely coherent with the results of Sabatino et al. [30], who stated that grafting combined with AMF significantly reduced GLY concentration in eggplant fruits.

## 4. Materials and Methods

### 4.1. Experimental Site, Plant Material, and Cultivation Conditions

The trial was accomplished in 2019 at Marsala, Sicily (37°44′54.7″ N 12°33′05.2″ E) during the winter–spring period. Three types of ‘Birgah’ F_1_ eggplant were tested: ungrafted, self-grafted, or grafted onto *S. torvum* rootstock. The grafting technique was performed as reported by Sabatino et al. [28] and Sabatino et al. [39] using a tube grafting method. On 12 February, at the end of the healing phase, plants were transplanted (2 plants m^−2^) in a polyethylene-covered tunnel. The soil was characterized by sand (<80%) at 8.5 pH and limestone (8.8%), 65 mg kg^−1^ of exchangeable K_2_O, 70 mg kg^−1^ of phosphorous, 2% of total nitrogen, and 10 t ha^−1^ of organic matter. The soil was mulched with black polyethylene film (20 µm) under which a drip irrigation system was installed. During the whole cultivation cycle, all plant needs were fulfilled based on the recommendations of Tesi [35].

### 4.2. Biostimulants Application

The two biostimulants, *Ascophyllum nodosum*-based extract (Stimplex^®^ Crop Biostimulant; Acadian Seaplants, Dartmouth, NS, Canada) (An) and *Trichoderma atroviride*, strain AT10 (Condor^®^, Verona, Italy) (Ta) were tested alone or combined (An+Ta). The biostimulant Stimplex^®^ was composed by 3-6% of protein/amino acids, 1% of lipids, 12-18% of alginic acid, 12–15% of fucose-containing polymers, 5–6% of mannitol, and 10–20% of other carbohydrates. The commercial product Condor contained 1 × 10^9^ UFC g^−1^ of T. *atroviride* strain AT10. Plants subjected to *Trichoderma atrovridae* strain AT10 were inoculated 24 h prior to transplanting, submerging the plantlet root system for 10 min at a rate of 1 kg ha^−1^. A second inoculation was repeated by watering 25 mL of inoculum per plant 35 days after transplanting (DAT) at a rate of 1 kg ha^−1^ (optimal dose).

For the non-microbial biostimulant, every 10 days, starting from 14 th DAT to the end of the harvest, the seaweed extract was supplied via foliar spray at a dosage of 0 and 3 (optimal dose) mL L^−1^, supplying 100 mL of solution per plant.

### 4.3. Plant Growth, Yield, and Yield Components

Growth and yield parameters were measured on all plants. Plant height (PH_50_), number of leaves per plant (NL_50_), and root collar diameter (RCD_50_) were recorded 50 DAT. After each harvest, total yield (TY) was measured, and the production was divided into marketable (MY) and non-marketable; total yield and marketable yield were expressed as kg plant^−1^. The number of marketable fruits (NF) was also recorded, and mean mass of marketable fruits was calculated (MF) and presented as g fruit^−1^.

### 4.4. Fruit Quality Traits

All quality parameters were determined on five fruits per replicate. All fruits were harvested at the same maturity stage (40 days after flower labelling). Fruit dry matter (FDM) was assessed by dehydrating fruits at 105 °C until they reached a constant weight. The values were expressed as percentages. Fruit firmness was evaluated through a penetrometer (model 53205, TR Snc., Forlì, Italy) by stinging the fruit in the middle part; its value was presented as newtons (N). Soluble solids content (SSC) was recorded on fruit juice via a refractometer and the values were reported as °Brix. The ascorbic acid (AA) concentration was measured as reported by Sabatino et al. [76]; values were expressed as mg 100 g^−1^ of fresh weight (fw). Chlorogenic acid (ClA) concentration was determined following the procedure of Stommel and Whitaker [77], with slight modification. The final quantification was accomplished by HPLC (Sigma-Aldrich, St. Louis, MO, USA) with an absorbance of 325 nm. Data were reported as mg 100 g^−1^ of dry weight (dw). Total anthocyanins content (TA) was measured using the method of Mennella et al. [78]. Briefly, the measurement was accomplished on 200 mg of lyophilized and powdered samples; then, for HPLC analysis, the flow rate was 0.8 mL min^−1^ and the absorbance was 0.1. The values were expressed as mg 100 g^−1^ dw. For fruit glycoalkaloids (GLY) determination, the extraction procedure of Birner et al. [79] was used. HPLC analysis was used, adopting solasonine and solamargine as standard. The measurement values were reported as mg 100 g^−1^ of dw.

### 4.5. Proteins and Mineral Profile in Fruit Tissue

All assessments on mineral profiles were performed on five fruits, casually chosen from each replicate. Nitrogen concentration in fruits was appraised via the Kjeldahl method, then the value was multiplied by 6.25 to obtain the protein content. Phosphorous (P) concentration was measured using the method of Fogg and Wilkinson [80]. Potassium (K), calcium (Ca), magnesium (Mg), zinc (Zn), copper (Cu), and iron (Fe) concentrations were determined through atomic absorption spectroscopy as described by Sabatino et al. [8] and Subramanian et al. [81]. Macro element concentrations (P, K, Ca, and Mg) were expressed as mg 100 g^−1^ dw, whereas microelements (Zn, Cu, and Fe) were reported as µg g^−1^ dw.

### 4.6. Experimental Design and Statistics

The 12 treatments were tested in a factorial experiment [three types of plant (ungrafted, self-grafted, or grafted) and four biostimulant treatments (control, An, Ta, or An+Ta)] using a completely randomized block design. Treatments were replicated three times and included 10 plants per replication, rendering 360 plants. The data were analysed using the SPSS v.20 software package via a two-way analysis of variance (ANOVA), setting type of plant and biostimulant as main factors. Means separation was accomplished using the Tuckey HSD test at *p* ≤ 0.05. Furthermore, a heat map displaying all the agronomic and qualitative traits of eggplant was realized using the online program package clustvis (https://biit.cs.ut.ee/clustvis/, accessed on 1 March 2022).

## 5. Conclusions

The continuous necessity of the horticultural sector to maximize yield and the concomitantly increasing consumer demand of premium quality foods pose a crucial challenge for researchers in their search for eco-friendly tools to enhance quantitative and qualitative crop traits. Biostimulants represent a key method in this exertion. In the current study, *A. nodosum* application elicited vigour traits, yield, and fruit quality of ‘Birgah’ F_1_ eggplant; however, the combined applications of *Trichoderma* and *A. nodosum*-seaweed extract further enhanced plant performance in terms of vigour features, productivity, nutritional and functional traits, and mineral profile. Moreover, the combination of both plant biostimulants and grafting onto *S. torvum* rootstock represents an encouraging, proficient, and environmentally friendly agronomic approach for increasing production and quality of eggplant grown in a protected environment.

## Figures and Tables

**Table 1:** Effect of plant type (T) and biostimulant (B) on fruit dry matter (FDM), fruit firmness, total anthocianins (TA), potassium (K), and iron (Fe) of ‘Birgah’ F_1_ eggplant.

Treatments	FDM (%)		Firmness (N)		ClA (mg 100 g^−1^ dw)		TA (mg 100 g^−1^ dw)		Proteins (g 100 g^−1^ dw)		K (mg 100 g^−1^ dw)		Fe (µg g^−1^ dw)	
T × B														
Ungrafted × Control	4.2	e	−46.2	a	735.6	d	7531.8	c	12.0	d	338.0	f	24.80	d
Ungrafted × An	4.3	e	−47.8	b	768.7	c	7607.8	b	13.9	c	353.0	e	25.37	c
Ungrafted × Ta	4.5	d	−46.8	a	739.0	d	7536.9	c	12.1	d	348.9	e	24.57	d
Ungrafted × An+Ta	4.5	d	−47.8	b	769.3	c	7606.6	b	13.9	c	360.9	d	25.40	c
Self-grafted × Control	4.3	e	−46.1	a	733.6	d	7614.8	b	11.9	d	346.2	e	24.87	d
Self-grafted × An	4.4	d	−48.0	b	768.8	c	7647.7	a	14.0	c	360.8	d	25.50	c
Self-grafted × Ta	4.4	d	−46.1	a	736.3	d	7615.7	b	11.9	d	358.9	d	24.57	d
Self-grafted × An+Ta	4.5	d	−48.2	b	769.8	c	7648.1	a	13.8	c	369.3	cd	25.67	c
Grafted × Control	5.3	c	−46.2	a	895.3	b	7228.5	e	15.4	b	363.6	d	31.80	b
Grafted × An	5.5	b	−48.5	b	987.5	a	7284.7	d	16.8	a	373.2	c	33.20	a
Grafted × Ta	5.8	a	−46.2	a	892.4	b	7228.4	e	15.4	b	380.3	b	31.50	b
Grafted × An+Ta	5.8	a	−49.7	c	995.7	a	7289.9	d	16.6	a	391.2	a	33.23	a
Significance														
T	***		***		***		***		***		***		***	
B	***		***		***		***		***		***		***	
T × B	**		***		***		***		***		**		**	

Means within the column and with the same letter are not significantly dissimilar according to Tuckey’s test (*p* ≤ 0.05); ** significant at *p* ≤ 0.01; *** significant at *p* ≤ 0.001.

**Table 2:** Effect of plant type (T) and biostimulant (B) on soluble solids content (SSC), ascorbic acid (AA), glycoalkaloids (GLY), phosphorous (P), calcium (Ca), magnesium (Mg), copper (Cu), and zinc (Zn) concentrations of ‘Birgah’ F_1_ eggplant.

Treatments	SSC (°Brix)		AA (mg 100 g^−1^ fw)		GLY (mg 100 g^−1^ dw)		P (mg 100 g^−1^ dw)		Ca (mg 100 g^−1^ dw)		Mg (mg 100 g^−1^ dw)		Cu (µg g^−1^ dw)		Zn (µg g^−1^ dw)	
T																
Ungrafted	4.3	a	6.55	b	76.67	a	513.40		104.04		17.68	a	2.68	b	9.61	b
Self-grafted	4.0	b	6.53	b	77.09	a	540.08		104.14		17.68	a	2.72	b	9.55	b
Grafted	3.9	c	7.13	a	46.64	b	513.98		104.41		15.75	b	3.57	a	10.57	a
B																
Control	4.1		6.58	b	71.73	a	502.12		104.42		16.27	d	2.82	b	9.68	b
An	4.1		6.90	a	61.84	b	526.36		104.20		17.17	b	3.17	a	10.14	a
Ta	4.1		6.52	b	72.06	a	501.32		103.93		16.86	c	2.81	b	9.68	b
An+Ta	4.0		6.94	a	61.57	b	560.14		104.23		17.86	a	3.14	a	10.13	a
Significance																
T	***		***		***		NS		NS		***		***		***	
B	NS		***		***		NS		NS		***		***		***	
T × B	NS		NS		NS		NS		NS		NS		NS		NS	

Means within the column and with the same letter are not significantly dissimilar according to Tuckey’s test (*p* ≤ 0.05); NS non-significant; *** significant at *p* ≤ 0.001.

## Data Availability

Not applicable.
